# Emotional Well-Being, Cognitive Load and Academic Attainment

**DOI:** 10.15694/mep.2020.000118.1

**Published:** 2020-06-04

**Authors:** William Atiomo

**Affiliations:** 1 University of Nottingham

**Keywords:** Cognitive, load, emotions, attainment, theory, extraneous, educational, academic, achievement.

## Abstract

This article was migrated. The article was marked as recommended.

This opinion piece examines some of the literature on the impact of emotional wellbeing on cognitive load and the impact this might have on educational attainment. It discusses cognitive load theory and outlines a brief history of its evolution to our current understanding. The literature on the degree to which negative emotions contribute to cognitive load is explored, followed by the association between cognitive load and educational attainment in the context of cognitive load theory. The article concludes by making a case for increased awareness, highlighting the need for more empirical research and briefly examining the role of some potential interventions to optimise cognitive load in the context of emotional wellbeing and their potential impact on educational attainment.

## Introduction

John is a fourth-year medical student at a medical school in the United Kingdom. He is currently doing his obstetrics and gynaecology placement. It is 23rd March 2020 and he is sat in the lounge in the house he shares with some friends, having a break, from revising for his end of year exams. He switches the television on and notices a news item on the British Broadcasting Corporation (BBC), announcing a UK wide lockdown as a result of the Coronavirus pandemic (COVID19). The University had also announced a cancellation of all face to face teaching and a moved to online teaching the week before. The medical school also advised fourth year students to go back home if possible, because of the pandemic. All of this is on his mind when he travels back home to London the following day on the 24 ^th^ of March. In addition to focusing on revising for his end of year exams, he has to mentally process the uncertainty caused by these events. People respond differently in a crisis and if this impacts negatively on his emotional wellbeing, will this in turn lead a higher likelihood of poor attainment at his obstetrics and gynaecology final examinations?

John will not be unique in his experience. Students all over the world are having to deal with the uncertainty of the current COVID19 pandemic with a probably varying nature of emotional responses. Even in the absence of a pandemic, many schools and higher education institutes internationally, have dedicated student welfare teams which aim to support students like John. These welfare teams see students with a range of issues some of which include mental health challenges (depression, anxiety, low mood, psychosis, personality disorders), disability, relationship problems, gender identity issues, financial challenges, learning difficulties (dyslexia, dyspraxia, attention deficit hyperactivity disorder (ADHD)), physical health needs (e.g. requiring surgery), bereavement, feelings of isolation and home sickness and students with a doubt about their future educational and professional careers. Many of these issues result in negative emotions which may negatively impact on academic attainment, which may in-turn further negatively impact on emotional wellbeing in a feedback loop.

Although a few theoretical frameworks through which negative emotions or poor emotional wellbeing might result in poor academic attainment have been advanced, empirical research directly testing this question is lacking. This article attempts to explore some of these questions by examining some of the literature on the impact of emotional wellbeing on cognitive load and the impact this might have on educational attainment. Research in this area may guide University faculty’s response to supporting John, and optimising his emotional wellbeing and attainment at his end of year exams. The concept of “wellbeing” has varying definitions, but this article focusses predominately on the emotional component of student well-being, which I have defined as the combination of positive affect (in the absence of negative affect) and general satisfaction with life. For the purposes of this article, I have also defined educational attainment as the results obtained following a summative assessment.

The article starts by discussing cognitive load theory and outlines a brief history of its evolution to our current understanding. The literature on the degree to which negative emotions contribute to cognitive load is explored, followed by the association between cognitive load and educational attainment in the context of cognitive load theory. The article concludes by making a case for increased awareness, highlighting the need for more empirical research and briefly examining the role of some potential interventions to optimise cognitive load in the context of emotional wellbeing and their potential impact on educational attainment.

## Wider Context

The intellectual performance and educational outcomes of students in educational institutes appears to be associated with student well-being ( Gutman and Vorhaus, 2012), (Yu, Shek and Zhu, 2018), ( Ayyash-Abdo and Sanchez-Ruiz, 2012), ( El Ansari and Stock, 2010). A public health England briefing for head teachers, governors and staff in education settings in published in 2014, states that research evidence shows a correlation between the wellbeing of students in schools and educational attainment (Public Health England, 2014).

Some studies have attempted to provide a conceptual or theoretical framework for the association between emotional wellbeing and educational attainment ( Gräbel, 2017). One such theory is the broaden-and-built theory of Barbara Fredrickson ( Fredrickson, 2004), which argues that the experience of positive emotions stimulates creative thinking and leads to a spread of attention and the broadening of behavioural resources (broaden-effect). In the theory, emotional wellbeing is directly linked to the experience of positive emotions. The enlargement of the behavioural and cognitive repertoire in turn promotes the development of long-lasting, effective coping strategies (built-effect) that support resilience and thus serves as a longitudinal resource against stress. There is however a paucity of empirical data on the effect of interventions that promote the health and wellbeing of students in schools on educational attainment ( Langford *et al.,*2014). This might be because of the lack of a clear theoretical framework upon which to investigate any associations between student wellbeing and educational attainment, although it might also be because of the complexity of confounding factors which might impact on the student’s “well-being”.

Cognitive load theory aims to provide a framework to educators on how information can be presented to students to optimize intellectual performance ( Sweller, Van Merriënboer and Paas, 1998). It has been argued that it is the single most important educational theory for teachers. The UK office for Standards in Education, Children’s Services and Skills (Ofsted) recently included cognitive load theory in its inspection framework for schools arguing that it is “a well-established theory, with over 30 years of research behind it, making it one of the best supported theoretical frameworks in education” ( Muijs, 2019).

Academic or educational attainment, appears to be strongly associated with the stimulation of meaningful learning by presenting information in a clear way ( Schneider and Preckel, 2017) which is consistent with the philosophy of cognitive load theory. There have however been no articles directly exploring whether the cognitive load theory might provide a useful theoretical framework upon which to investigate any associations between student’s emotional wellbeing and educational attainment. A recent paper published last year ( Hawthorne, Vella-Brodrick and Hattie, 2019) explored how student well-being may act to reduce cognitive load. However, it did not directly address the issue of educational attainment.

## Cognitive Load Theory

Cognitive load theory is based upon three components (1) cognitive architecture explained through evolutionary principles, (2) the division of cognitive load into three categories and (3) instructional effects.

The cognitive architecture component of cognitive load theory is based on the premise that during the process of learning, new information is initially stored in short term or working memory and later transferred into long term memory for storage and future retrieval. The theory states that while working memory is limited in both capacity and duration, allowing for maintaining and processing only a few pieces of information at any given time ( Young *et al.,*2014), the capacity of long term memory is unlimited (Sweller, Van Merriënboer and Paas, 2019). With respect to instructional design or teaching and effects, it argues that if the total cognitive load associated with learning a task or skill exceeds the capacity of the available working memory, additional information the learner is exposed to, is unavailable for long term storage and future retrieval and learning, therefore attainment and performance may be impaired. On the other hand, a cognitive load that is too low may cause boredom.

Three types of cognitive load have been defined; *intrinsic, extraneous and germane.* Intrinsic cognitive load refers to the complexity or nature of the information being learnt. This includes task complexity (the number of information elements that must be simultaneously processed in working memory) and is dependent on learner expertise (the sophistication of the learner’s schemas or prior knowledge, related to the task). Intrinsic cognitive load is therefore a function of the task-learner interaction ( Haji *et al.*, 2015) and depends on how information is presented to students. For example, research in helicopter pilots showed that cognitive load was highest when tactical information was displayed as text, midlevel when displayed as numeric, and lowest when displayed in graphical format ( Dominessy *et al.*, 1991).

Extraneous cognitive load refers to factors external to the material or information being learnt which increase cognitive load. Some examples include suboptimal instructional design or the way in which the information is presented (e.g. requiring students to look at power point slides as well as a research article during a lecture and unnecessarily having to search for information). Other examples include external distractions (e.g. noise) and language of instruction (requiring knowledge of a non-native or second language to understand the information). A key focus of this article however is on the internal distractions (e.g. negative emotions, worries or thinking about external or personal issues, competing demands for time and self-induced time pressure) ( Sewell *et al.* *,* 2019) which may also contribute to extraneous load.

Germane cognitive load refers to the way in which schema or patterns obtained from prior learning and experience impact on the new material being learnt, or the cognitive resources (acquisition and automation of schemas stored in long-term memory) invested in dealing with intrinsic cognitive load, that contributes to learning. It is recommended that instructional designers promote germane cognitive load by for example, including exercises to activate prior knowledge at the start of a teaching session. Other suggested methods to promote germane load included situational awareness training ( Saus *et al.,*2006), ( Saus, Johnsen and Eid, 2010), self-explanation, asking clarifying questions, and/or confirming one’s understanding ( Young *et al.,*2016), greater teacher engagement with learners ( Sewell *et al.,* 2017) and careful design of feedback practices (Lee. and Lee, 2018). Germane cognitive load is related to the profile of the learner, for example, a graduate entry medical student who has previously worked as an optician may have their germane cognitive load promoted during a lecture on diseases of the eye compared to another medical student who has had no prior work experience in ophthalmology or the opticians.

Cognitive load theory, advocates for instructional designs that maximise learning by reducing extraneous load, optimising germane load and managing intrinsic load (Sweller, Van Merriënboer and Paas, 1998). In practice, whilst managing intrinsic cognitive load is largely dependent on the instructor/instructional design, extraneous cognitive load as highlighted above, may be increased by emotional upset and worry, such as may have been present in John, the medical student whose story was outlined at the start of this article, in response to the news of the COVID19 pandemic.

## Evolution of cognitive load theory

One of the early proponents of the cognitive load theory was John Sweller, an Australian cognitive psychologist in the 1980s. In a paper published in Cognitive Science ( Sweller, 1988) within the context of 15-16 year old, Mathematics students, Sweller argued that the move by educationalists fostered by Dewey towards discovery based learning or problem based learning was not associated with a commensurate knowledge of its characteristics and consequences. He argued that contrary to the state of affairs at the time, many forms of problem solving during the teaching of mathematics, interfered with learning by increasing cognitive load. At this stage, the different types of cognitive load had not been defined and the paper was primarily focused on instructional design within mathematics and the possible impact of emotions were addressed.

To support Sweller’s theory, research was also presented in which subjects trying to solve a maze problem who had been shown the goal position failed to induce the essential structural features of the problem which sometimes prevented them from even solving relatively simple problems. In a study of 24 maths students aged 15 to 16 years, in a Sydney (Australia) high school, they were asked to solve 6 problems involving the use of sine, cosine and tangent ratios. Students in the study group were told that their major task was to solve the problem. A conventional and non-specific group (goal free) of 12 subjects each were used. Students in the goal free group required a marginally less time to solve the 6 problems although this did not quite reach statistical significance. This group however also made significantly fewer angle position, side value, side position and solution errors compared to the conventional group. Sweller argued that this was evidence of a higher cognitive load during conventional problem solving relative to the non-specific goal group and that more excess cognitive capacity appeared to be available after solving a non-specific goal problem compared to a conventional one. Within the context of learning, Sweller argued that the problem solving/goal oriented/means ends approach to learning, limited the cognitive processing capacity for acquiring new schemas or knowledge. Again, this did not address the possible impact of emotional wellbeing on cognitive load.

Sweller’s cognitive load theory has however not been without its controversies. In an article by Michael Pershan ( Pershan, 2016), the evolution of the cognitive load theory is described including how concepts such as intrinsic and extraneous cognitive load were introduced to refer to the cognitive load intrinsic to the task itself which were not modifiable and extraneous (modifiable) cognitive load which referred to the format in which the material to be learnt was presented. Extraneous cognitive load included the cognitive load associated with finding the goal or solution to a problem and was initially thought to be undesirable. The goal of instructional design being to limit the extraneous cognitive load as much as possible so that the capacity available in working memory could be freed up to facilitate learning. The article by Pershan also stated that the concept of germane cognitive load was introduced later to reflect the cognitive activity of analysing new knowledge in the context of existing schemas. Perhan’s article also outlined how Sweller’s view on the need to avoid problem solving / goal oriented teaching also evolved and that in Sweller subsequently argued that in some contexts, perhaps where the teaching was directed at a cohort with a reasonable level of expert knowledge and schemas, a high cognitive load and a goal focused approach to teaching and learning was not unreasonable. It was argued that in the teaching of complex skills such as riding a bicycle, it was not realistic to apply the cognitive load theory as all the components required to learn such a skill were interdependent. It was not possible for example to reduce cognitive load by teaching a student to steer a bicycle first, before learning to pedal, or balance. Within the context of medicine, this argument may also apply to the teaching of practical procedures such as laparoscopic surgery. The concept of the “Expert Reversal Effect” was also introduced; where using cognitive load theory principles that were effective in novices were counterproductive in experts. These developments although highlighting the concept of extraneous cognitive load, never really focussed on the possible impact of emotional well-being on cognitive load.

## Measuring cognitive load

Measuring cognitive load currently relies broadly on four methods; subjective self-rating questionnaires of how much mental energy was invested in a learning exercise, secondary task measures, physiological changes and observer ratings. The first rating scale measure of cognitive load was introduced in the early 1990s by Fred Paas ( Sweller, 2018). Other rating scales including the NASA-TLX and several investigator-developed scales have also since been were utilized ( Sweller, 2018) as well. Before the introduction of these scales, John Sweller states that the only attempt to provide an independent indicator of cognitive load was to use computational models with quantitative differences between models used as cognitive load proxies and that the Paas scale continues to be the most popular measure of cognitive load ( Sweller, 2018). The challenge with these measures of cognitive load in any planned studies of emotional wellbeing is the possible confounding effect of the emotional state of the subject being studied.

The use of physiological indicators to measure cognitive load, include heart rate and respiratory rate variability, electroencephalogram, electromyography, eye tracking, pupil diameter, blink frequency, serum adrenaline levels, brain imaging, and skin conductance ( Schmeck *et al.,* 2015) as these are thought to indirectly reflect working memory demands ( Haji *et al.,* 2015). Performance on secondary tasks (e.g. memory or stimulus detection tasks) have also been used and are thought to reflect ‘spare’ working memory capacity not consumed during training. Again, these physiological measures of cognitive load may be confounded by the physiological responses induced by various emotional states.

A review of measurements of cognitive load ( Naismith and Cavalcanti, 2015) found that multiple studies employed more than one method to measure cognitive load or mental work-load and often demonstrated at least some agreement among measurement methods. However, the broad heterogeneity of included studies prevented drawing conclusions about any particular cognitive load measurement technique as superior.

## Emotional wellbeing, academic attainment and cognitive load theory

Evidence to enable a critical appraisal of the argument in this article, as to whether cognitive load theory could provide a credible theoretical basis on which to explore any associations educational attainment and student emotional wellbeing is challenging given the range of potential confounding effect of the various theories of education and the confounding effects of various emotional states on the measures of cognitive load as highlighted above. These theories of learning encompass cognitive, affective, social, environmental and metacognitive theories. Student motivation (which may be dependent on a student’s emotional state) and the language of instruction (primary or secondary), may also theoretically affect working memory and cognitive load. The association between emotions and cognitive load theory is however a useful place to focus in exploring any associations between emotional well-being and attainment. The second key area to examine is studies linking cognitive load with attainment.

### Emotions and cognitive load

Anxiety is defined as a motivational state that exists when there is a high level of perceived threat to that individual ( Derakshan and Eysenck, 2009) and under such circumstances, state anxiety has been found to impair cognitive performance, including processes such as working memory and allocation of attentional resources. Anxiety may therefore increase cognitive load. The relationship between anxiety and cognitive load is however complex because, the impact of allocation of attentional resources and cognitive load during anxiety may vary dynamically with stages of processing. An initial orientation to threatening information may be characterised by vigilance, however, once threatening information has captured attention, threatening stimuli can either be avoided (fast disengagement) or can continue to capture attention (difficulty disengaging). This complexity of the relationship between anxiety and cognitive load is also highlighted by the fact that increased cognitive load and working memory, can reduce anxiety, an effect which is thought to be boosted by exercise ( Lago *et al.,* 2019).

To add to this complexity, an emotional state of shame was found in one study of 49 medical students to improve learning by processing germane load ( Hautz *et al.*, 2017). Students being trained on breast examinations with a standardized patient experienced more shame during training, spent more time with the patient and documented more breast lumps. Furthermore, it has been argued that a high workload and by inference cognitive load, is an important but not a critical factor in the development of stress symptoms and that it is quite possible to work hard in difficult and complex tasks, even under unfavourable conditions, without cognitive strain. Whereas stress on the other hand is typically is characterized by inefficient behaviour, over reactivity, and the incapacity to recover from work ( Gaillard, 1993).

A recent review article ( Plass and Kalyuga, 2019) on emotions and cognitive load theory described four ways of considering emotions in cognitive load theory. These four ways include the impact of emotions on extraneous cognitive load, intrinsic cognitive load, memory and motivation. With respect to extraneous cognitive load, a suppression hypothesis was described ( Ellis and Ashbrook, 1988). Negative emotions can either lead to the allocation of resources to extra-task processing, i.e., thinking about one’s emotional state, or to task irrelevant processing, i.e., processing information not related to the learning outcome both of which will reduce the capacity of the available working memory for learning new material. However, this may also increase the amount of effort invested in a task ( Baddeley, 2012), ( Eysenck and Calvo, 1992). Stereotype threat may involve similar mechanisms, in which members of a negatively stereotyped social group, experience anxiety about their performance, and processing of the resulting emotions may result in reduced outcomes ( Steele and Aronson, 1995). This may or may not explain some of the data and concerns about differential attainment of students from a minority socioeconomic or ethnic group. However, this is a complex area as low socioeconomic status has been thought to create higher risk for stress, adversity, and in turn, psychiatric disorders, from a social causation perspective.

Emotions may also have an impact on intrinsic cognitive load. In medicine, students need to learn to deliver bad news to patients or to deal with the death of a patient. In these cases, emotional regulation is part of the learning objectives and processing or regulating emotions is therefore essential, making it a source of intrinsic cognitive load ( Plass and Kalyuga, 2019). In studies on simulated patient death, students who experienced higher levels of emotions reported higher cognitive load and showed lower learning outcomes on the performance tasks ( Fraser *et al.,* 2014).

With respect to memory, working memory may be affected by emotion in different ways. These include enhanced encoding, broadening or narrowing of resources, and mood-dependent encoding and retrieval. For example, positive emotions may signal that an individual’s needs are taken care of, allowing for other goals and needs to be addressed ( Carver, 2003). This is thought to have a broadening effect that may increase the amount of cognitive resources available for learning ( Fredrickson, 1998), ( Fredrickson, 2001). Negative emotions on the other hand, may indicate that particular needs or goals are not yet sufficiently addressed. This may have a narrowing effect that decreases the amount of cognitive resources available for learning ( Fredrickson, 2003). Finally, positive emotions may also affect motivation to increase cognitive effort which will result in increased learning and negative emotions in disengagement from the learning process. The relationship between emotions and motivation to learn is however complex as unpleasant or negative emotions may lead to a higher investment in cognitive effort compensating for any short term increases in cognitive load and reduction in working memory during a specific learning exercise.

In addition to the complexity of the relationship between emotional states and cognitive load as highlighted above, as previously highlighted, the use of physiological indicators also potentially poses a challenge in exploring the link between cognitive load theory and student wellbeing, as these physiological measurements of cognitive load can be influenced by different unpleasant emotional states and stress. These physiologic measurements of cognitive load also do not differentiate between the different types (intrinsic, extraneous and germane).

The complexity of possible research into investigating the association between emotions and cognitive load theory is also illustrated by the possible impact of fatigue. Anxiety worry and other negative emotions may cause lack of sleep leading to fatigue which can affect both intrinsic and extraneous cognitive load ( Sewell *et al*., 2017). Social-evaluative or stereotype threat is also a central aspect of stressors that have been found to elicit a cortisol response which might confound physiological measurements of cognitive load in any study designs although there does not appear to be evidence that increased difficulty, or cognitive load, contributes to greater cardiovascular or cortisol responses to stressors ( Haji *et al.,*2015).

### Cognitive Load Theory and Attainment

Another key area to explore to address the question in this article, which aims to explore and associations between emotional well-being and attainment, is exploring the evidence that the use of cognitive load theory improves attainment. The complexity of confounding variables however makes it challenging to address this question with precision. However, given the suppression hypothesis ( Ellis and Ashbrook, 1988), outlined above with respect to extraneous cognitive load and negative emotions such as depression, a useful place to focus would be investigating the association between extraneous cognitive load and educational attainment.

Studies have however found that test anxiety is not associated with academic performance but correlates with effort/reward imbalance ( Hahn *et al.,* 2017). Although there is thought to be some relationship between educational attainment and psychiatric disorders ( Peyrot *et al.,* 2015), ( Fletcher, 2010), ( Erickson *et al.,* 2016) it is thought that this might be a proxy for socioeconomic status which is associated with low educational attainment, with a low socioeconomic status thought to create higher risk for stress, adversity, and in turn, psychiatric disorders.

Although John Sweller argues that the Paas scale has repeatedly indicated that instructional designs hypothesized to decrease cognitive load as measured by the scale, increase performance test scores ( Sweller, 2018), there have not been any empirical studies directly testing the hypothesis that reducing extraneous cognitive load improves examination performance.

In a literature search of the ProQuest and PubMed databases on 5 ^th^ May 2020, using the terms “cognitive load theory and examination results”, “cognitive load theory and examination attainment”, “extraneous cognitive load and examination results” and “extraneous cognitive load and examination attainment”, no empirical studies were identified, directly investigating the hypothesis that reducing extraneous cognitive load improved examination attainment.

Other search terms included “Cognitive load theory” and “academic achievement”, “academic performance”, “examination performance”, “academic attainment”, “attainment”, “educational attainment”, “performance”, or “examination attainment”; “Cognitive load” and “performance”, “examination performance” or “academic performance”; “psychological wellbeing” and “examination performance” or “academic achievement”. “Emotional wellbeing” and “academic achievement”, “academic performance”, “academic attainment”, “examination performance”, “examination attainment” or “examination achievement”, also did not yield any empirical studies directly investigating emotional wellbeing, extraneous cognitive load and educational attainment. One systematic review of 48 studies, found inconsistent correlations between cognitive load and learning and argued that this might have been related to issues of validity in cognitive load measures ( Naismith and Cavalcanti, 2015).

There is therefore currently no direct empirical evidence that increased extraneous cognitive load as a result of poor emotional wellbeing, impairs educational attainment. There is therefore currently no empirical data on which to base any strategies to improve academic attainment, by reducing any extraneous cognitive load caused by the emotional upset secondary to the COVID19 pandemic in John (the medical student discussed at the start of this article).

## Possible future research directions & conclusion

Any future studies to attempt to obtain any data to validate the research question; “does emotional wellbeing influence academic attainment, by affecting extraneous cognitive load” will in the first instance, have to be a prospective observational study as a randomised controlled trial will not be feasible to establish associations. There will however be several challenges to address, including a need to control for the possible confounding effect of various emotional states on measurements of cognitive load, the validity of measurements obtained at a single time point (in contrast with serial data collection), issues of student confidentiality, agreeing a validated measure of extraneous cognitive load and an agreement on which measure of academic attainment to use (e.g. module mark, end of year mark or final year degree classification). It will also be important to address possible confounding factors which may affect attainment, including, historic academic attainment, gender, socio-economic group and language of instruction. I do however think designing and delivering a study to address this question is possible though. Perhaps a prospective study, in which students are administered a validated questionnaire which measures their emotional state as well as a questionnaire to measure total and extraneous cognitive load can be conducted. These questionnaires could be administered after a lecture at the start of the academic year, halfway through the year and at the end of the academic year and the results correlated with the end of year examination results. The results of such a study would be helpful in subsequent studies, investigating the effectiveness of interventions to improve academic attainment, by reducing any extraneous cognitive load, in the context of randomised controlled trials. Some interventions which might be interesting to investigate, include the impact of cognitive or behavioural strategies on emotions, extraneous cognitive load and attainment.

Support provided to John (the medical student upset by the COVID19 pandemic, discussed at the start of this article) and other students in a similar situation, to improve their emotional wellbeing and academic attainment can therefore be based on the findings of empirical research.

## Take Home Messages

• A student’s poor emotional wellbeing, may reduce educational attainment, by increasing extraneous cognitive load during learning.
• Cognitive load theory may provide a framework to investigate the association between student’s emotional wellbeing and educational attainment.
• There is a need for empirical research to investigate whether student’s emotional wellbeing, influences educational attainment by affecting, extraneous cognitive load.
• There is a need for empirical studies investigating the impact of cognitive or behavioural strategies on emotional wellbeing, extraneous cognitive load and educational attainment.

## Notes On Contributors

William Atiomo (MBBS, DM, MA, FRCOG, SFHEA) is clinical associate professor in gynaecology and clinical sub-dean in the school of medicine, University of Nottingham ( https://orcid.org/0000-0002-5027-3504).
