# Supplementary material for: Emotional Well-Being, Cognitive Load and Academic Attainment
Source: MedEdPublish (2016). 2020 Jun 4;9:118. [Version 1] doi: 10.15694/mep.2020.000118.1 (PMC10702652; doi:10.15694/mep.2020.000118.1)
Supplement: Peer review history

# Reviewer report: Reviewer response for version 1

**DOI:** 10.21956/mep.20085.r30925

**Published:** 2020-06-26

**Authors:** Valerie Farnsworth

**Affiliations:** 1 University of Leeds

This review has been migrated. The reviewer awarded 3 stars out of 5 While I appreciate the overall argument being made in this article, I am concerned that the emphasis is too strongly on ‘empirical research directly testing’ the question of cognitive load theory and its impact on well-being. The implication is that we need this evidence-base before we can claim that cognitive load is impacting emotions. However, in my mind, the authors present a good deal of research that gives us reasonable justification to refine our interventions, accounting for concerns around cognitive load. I would dispute the claim that cognitive load intrinsic to a task is ‘not modifiable’ and this sort of thinking of knowledge as a single ‘truth’ needs to be disrupted (e.g. recognise the social construction of knowledge). In fact, such disruption is linked to calls to decolonise the curriculum. Even extraneous cognitive load will be socially constructed. Two of our students researched the role of music in medical students (Glen Davies and Joslan Scherewode) and found that listening to music could be an extraneous distraction but depending on the music, could also support focused studying and engagement in the task at hand as well as de-stressing. I appreciate that cognitive load can be impacted by emotions and educators should consider ways to minimise negative emotions if these are not intrinsic to the learning outcomes being addressed (e.g. to make clinical decisions in stressful contexts). I particularly appreciate the recognition that there may be such negative emotions that are associated with minority status (or for that matter, any other social factor such as gendered discourse evoked in the social learning context). However, I would caution against assumptions that socioeconomic status is automatically indicative of psychiatric disorders, as this neglects the ways adversity can build resilience and commitment, as another student of ours, Sonal Mistry, found in her study of the journey to medicine for a sample of MBChB students who met Widening Participation criteria. The article seems to be underpinned by a computational model of learning and the field of education has been disrupted by social theories of learning, such as social constructionism. Concepts missing from this account are self-directed learning, growth mindset, distributed cognition and metacognition. I would argue that rather than more experimental studies we need to explore meaning making and explore intersections between cognitive load theory and social theories of learning. Practically speaking, given the variability and complexity discussed, particularly in relation to emotions and cognitive load, would it not be best to equip students with skills to identify and recognise when external stressors or emotions are having a negative impact or whether it is promoting their learning? The research in this article would be useful resources for students in their own self-assessment and reflections. In addition, we need more research that helps us think about the way the social learning environment is implicated in outcomes observed and I believe a constructivist approach is the best tool for that analysis. Further experimental research may lead to more debates and what we need now is action.

Reviewer Expertise:

NA

No decision status is available

# Reviewer report: Reviewer response for version 1

**DOI:** 10.21956/mep.20085.r30923

**Published:** 2020-06-12

**Authors:** Tharin Phenwan

**Affiliations:** 1 University of Dundee

This review has been migrated. The reviewer awarded 4 stars out of 5 Thank you for this remarkable perspective. It is interesting to know more about Cognitive load theory and how scarce the evidence is. We need to do a lot more especially in this new normal so that we can come up with better strategies to support the students.This will be of interest of those who want to use education theories to improve their curriculum or teaching modules

Reviewer Expertise:

NA

No decision status is available

# Reviewer report: Reviewer response for version 1

**DOI:** 10.21956/mep.20085.r30924

**Published:** 2020-06-11

**Authors:** Hebat Allah A. Amin

**Affiliations:** 1 Department of Pathology, Helwan University, Faculty of Medicine

This review has been migrated. The reviewer awarded 4 stars out of 5 An interesting article. I would recommend designing a figure summerizing the components of the cognitive load theory and factors affecting. I think it would be helpful for deeper understanding.

Reviewer Expertise:

NA

No decision status is available

# Reviewer report: Reviewer response for version 1

**DOI:** 10.21956/mep.20085.r30927

**Published:** 2020-06-10

**Authors:** Nagwa Hegazy

**Affiliations:** 1 Menoufia university

This review has been migrated. The reviewer awarded 4 stars out of 5 Authentic thoughtful work.I liked the cognitive load theory explanation, types of cognitive load and measurements.I had enjoyed reading the possible relation between emotional well being, cognitive load and educational attainment.I think more work is needed in that area for better understanding and coping strategies.Thanks!

Reviewer Expertise:

NA

No decision status is available

# Reviewer report: Reviewer response for version 1

**DOI:** 10.21956/mep.20085.r30926

**Published:** 2020-06-07

**Authors:** Laurel Fick

**Affiliations:** 1 Ascension St. Vincent Hospital

This review has been migrated. The reviewer awarded 4 stars out of 5 Innovative and thoughtful discussion on the role of cognitive load in educational attainment. In an era of increasing pressures on simultaneous care quality and efficiency, the role of cognitive demands for a learner deserve this thoughtful discussion. Thank you!

Reviewer Expertise:

NA

No decision status is available
